# A novel phase-difference transcranial alternating current stimulation system enables precise dual-site neuromodulation

**DOI:** 10.3389/fnins.2026.1796456

**Published:** 2026-04-10

**Authors:** Ruiren Wu, Ying Feng, Jiali Wu, Jingjing Zhang, Wei Feng, Cong Wang, Chunlei Shan

**Affiliations:** 1 School of Rehabilitation Science, Shanghai University of Traditional Chinese Medicine, Shanghai, China; 2 Hongqiao International Institute of Medicine, Tongren Hospital, Shanghai Jiao Tong University School of Medicine, Shanghai, China; 3 Yueyang Hospital of Integrated Traditional Chinese and Western Medicine, Shanghai University of Traditional Chinese Medicine, Shanghai, China; 4 Department of Rehabilitation Medicine, Tongren Hospital, Shanghai Jiao Tong University School of Medicine, Shanghai, China; 5 Yuanshen Rehabilitation Institute, Shanghai Jiao Tong University School of Medicine, Shanghai, China; 6 Shanghai Key Laboratory of Flexible Medical Robotics, Tongren Hospital, Institute of Medical Robotics, Shanghai JiaoTong University, Shanghai, China

**Keywords:** amplitude stability, multiple brain regions, phase-difference stimulation, rehabilitation engineering, transcranial alternating current stimulation

## Abstract

Precise modulation of large-scale brain networks requires neuromodulation technologies capable of delivering frequency-locked stimulation with accurate and stable inter-regional phase control. However, conventional transcranial alternating current stimulation (tACS) systems generally lack robust dual-channel phase regulation and are rarely validated under realistic biological impedance conditions. Here, we present a novel phase-difference tACS system (PD-stim) designed to deliver programmable, high-precision phase offsets between stimulation targets. We performed a comprehensive engineering and *in vivo* validation of PD-stim, assessing biological impedance stability, waveform fidelity, amplitude stability, and phase-delivery accuracy. Impedance measurements obtained from the medial prefrontal cortex and hippocampus of rats demonstrated stable frequency-dependent profiles during stimulation. Benchmark comparisons against a clinically approved tACS device revealed comparable waveform fidelity and amplitude stability under both a standardised resistive load and *in vivo* recording conditions. Using simultaneous dual-channel oscilloscope recordings, PD-stim consistently generated stable sinusoidal waveforms with high phase-delivery accuracy across theta (8 Hz), beta (20 Hz), and gamma (40 Hz) frequency bands, under both biological and resistive conditions. Together, these results establish PD-stim as a precise, stable, and biologically robust dual-site neuromodulation platform that overcomes key technical limitations of existing tACS systems. This work provides a rigorously validated engineering framework for future mechanistic investigations of phase-specific modulation in distributed brain networks, while not addressing functional or therapeutic outcomes.

## 1 Introduction

Non-invasive brain stimulation has emerged as a promising approach for modulating neural activity and promoting functional recovery in neurological disorders, including stroke, traumatic brain injury, and age-related cognitive decline (Rektorová et al., 2025; Polanía et al., 2018). Among these techniques, transcranial alternating current stimulation (tACS) has attracted increasing interest due to its ability to interact with endogenous neural oscillations and influence network-level synchronisation in a frequency-specific manner (Helfrich et al., 2014; Wischnewski et al., 2023).

Neural communication across distributed brain regions depends not only on oscillatory power but critically on inter-regional phase relationships, which regulate information flow, synaptic plasticity, and cognitive processing (Arnulfo et al., 2020; Wang et al., 2021; Wang et al., 2022). Disruptions in phase synchrony are a hallmark of neurological injury and ageing-related disorders and are closely associated with impaired motor and cognitive function (Al-Ezzi et al., 2024; Rogala et al., 2024; Tscherpel et al., 2025). Studies have shown that simultaneous entrainment of neural activity to externally applied oscillatory electric fields enables controlled modulation of phase relationships among intrinsic neural oscillations, allowing systematic investigation of their functional significance (Fell and Axmacher, 2011; Fries, 2015; Hanslmayr et al., 2016; Haslacher et al., 2024).

These insights have motivated growing interest in phase-specific neuromodulation strategies aimed at restoring pathological network dynamics rather than merely increasing or suppressing local activity. To date, studies have largely focused on two principal stimulation configurations: in which the alternating currents applied to the two electrodes are either phase-aligned (0° phase difference) or anti-phase (180° phase difference) (Alekseichuk et al., 2019; Elyamany et al., 2025; Preisig et al., 2019), a binary paradigm widely established to modulate cognitive performance and frontoparietal network dynamics.

Despite this conceptual advance, many existing tACS implementations have not been rigorously validated for inter-channel phase delivery under realistic loading conditions, and multi-electrode stimulation can produce complex spatiotemporal electric-field dynamics that make phase control non-trivial (Lee et al., 2023). To address these limitations, we developed a novel phase-difference tACS system (PD-stim) capable of delivering frequency-locked, dual-channel stimulation with high-resolution phase control. Unlike conventional systems, PD-stim was specifically engineered to (i) maintain waveform fidelity and amplitude stability under variable biological impedance, and (ii) achieve accurate and reproducible inter-channel phase offsets across multiple frequency bands relevant to motor and cognitive rehabilitation.

In the present study, we performed a comprehensive engineering and *in vivo* validation of the PD-stim platform using a hierarchical framework designed to bridge technical development with clinical translation. We systematically evaluated four critical performance metrics—impedance stability, waveform fidelity, amplitude consistency, and phase-delivery accuracy—progressing from standardized resistive loads to implanted electrodes in the medial prefrontal cortex (mPFC) and hippocampus of rats. This stepwise validation strategy was specifically engineered to address the technical challenges of neuromodulation under dynamic biological conditions: first, confirming that the Howland current source maintains constant-current output across fluctuating tissue impedances (0–10 kΩ); second, verifying that pure sinusoidal waveforms are delivered with minimal distortion, ensuring precise oscillatory entrainment; third, demonstrating long-term amplitude stability over clinically relevant stimulation durations (20 min); and fourth, establishing that dual-channel phase offsets are controlled with 0.1° resolution and >97% accuracy under *in vivo* conditions. By benchmarking PD-stim against a clinically approved tACS device and validating performance under biological neural load in a functionally connected circuit (mPFC-hippocampus), we aim to establish a robust technological foundation for future phase-targeted neuromodulation therapies in neurorehabilitation, where precise control over inter-regional phase relationships may enable circuit-specific treatment of neurological and psychiatric disorders.

## 2 Methods

### 2.1 PD-stim device engineering

#### 2.1.1 Phase-difference tACS (PD-stim) device development

The stimulation framework incorporates a configurable parameter input module enabling user control over waveform type (sine or square), peak output current, stimulation frequency, programmable inter-channel phase offsets, and stimulation duration. A dual-channel digital-to-analogue converter (DAC) architecture provides high-precision waveform generation using a digital lookup-table strategy. Generated voltage signals are subsequently converted to stable current output through a high-voltage Howland current source, based on an optimized Howland current pump design to ensure excellent linearity and output stability.

The stimulation electrode output adopts a shared-electrode configuration, in which stimulation and recording share identical skull screw electrodes, reference, and ground structures, effectively minimising inter-channel crosstalk. A real-time waveform monitoring module continuously samples output signals. When connected to a standard resistor, waveform fidelity is evaluated; when connected *in vivo*, biological impedance is dynamically monitored. Based on these measurements, the system automatically determines waveform quality and biological safety status and can autonomously terminate stimulation if required.

#### 2.1.2 Core phase-control algorithm

High-precision sinusoidal phase regulation is achieved using a digital lookup-table algorithm with 0.1° phase resolution. Firmware stores a sinusoidal table containing 3,600 sampling points corresponding to a full 360° waveform cycle. Phase values are generated according to:

$$\text{sinPhaseValue}[i]=A\cdot \sin(\frac{2\mathrm{\pi i}}{3600})$$

where A defines waveform amplitude. By independently adjusting the lookup start index of the two DAC channels, precise inter-channel phase offsets are obtained, with each index increment corresponding to 0.1° of phase shift, enabling stable maintenance of predefined phase differences throughout stimulation.

#### 2.1.3 Hardware configuration

The stimulator generates two independent stimulation waveforms, each supporting ±75 V peak voltage. Output current precision is calibrated using a resistor network, achieving 12-bit DAC accuracy. A USB Type-C communication interface connects the stimulator to a personal computer, where custom software allows full parameter customization and real-time transmission via a dedicated serial protocol.

Two stimulation electrodes connect the stimulator to implanted or multi-channel electrode assemblies. A dual-channel oscilloscope serves as an external monitoring system for waveform verification. To minimise electrical interference, both the stimulator and oscilloscope are powered by independent rechargeable lithium battery systems, ensuring electrical isolation and noise stability.

### 2.2 Biological validation

#### 2.2.1 Rationale for *in vivo* biological validation

To determine whether the engineered PD-stim platform can seamlessly translate its bench-top precision to highly dynamic physiological environments, we developed a comprehensive in vivo validation strategy. The medial prefrontal cortex (mPFC) and hippocampus (HPC) form a functionally coupled network characterized by prominent low-frequency oscillatory synchronization (e.g., theta phase coupling), making it an ideal, biologically rigorous testbed. By deploying our system within this distributed circuit, we aimed to ascertain whether its core technical innovations—specifically, the high-compliance Howland constant-current regulation, the 0.1° high-resolution phase-control algorithm, and the shared-electrode architecture—could collectively maintain stringent waveform fidelity and precise inter-regional phase offsets against the continuously fluctuating, frequency-dependent impedances inherent to living neural tissue.

#### 2.2.2 Animals

Male and female Sprague–Dawley (SD) rats (5–12 weeks old) were obtained from the Experimental Animal Center of Shanghai Jiao Tong University. Animals were housed in groups of 2–4 per OptiRAT cage under a 12 h light/dark cycle, with ad libitum access to food and water. All experimental procedures were conducted during the light phase. All procedures adhered to the institutional guidelines for the care and use of laboratory animals of Shanghai Jiao Tong University and were approved by the Institutional Animal Care and Use Committee of Shanghai Jiao Tong University (approval number: A2024243-002).

#### 2.2.3 Surgical implantation of stimulation and monitoring electrodes

Custom-made multi-electrode arrays (Bio-Signal Technologies, Nanjing, China) were stereotaxically implanted into the medial prefrontal cortex (mPFC) and hippocampus (HPC) to enable simultaneous dual-site tACS stimulation using the PD-stim device and concurrent current waveform monitoring via an external oscilloscope. Rats were anesthetised with 1–3% isoflurane (RWD Life Sciences, Shenzhen, China, R510-22) and secured in a stereotaxic apparatus (RWD Life Sciences, Shenzhen, China, 68,025). Body temperature was maintained at 37 °C using a heating pad throughout the procedure. Prior to implantation, all electrode wires were coated with DiI fluorescent dye (Invitrogen, CAT #: V22885) to enable subsequent histological verification of electrode tip locations. Electrodes were unilaterally implanted using the following stereotaxic coordinates (mPFC: AP = +0.4 mm; ML = +2.76 mm; DV = −3.8 mm. HPC: AP = +5.4 mm; ML = −5.4 mm; DV = −4.8 mm) relative to bregma. A ground wire was positioned beneath the skull and above the dura through a small hole drilled on the contralateral hemisphere. At the end of the surgery, animals received carprofen (5 mg/kg, Shanghai YuanyeBio, Shanghai, China, S71066-5 g) and Augmentin (50–100 mg/kg, MeilunBio, Dalian, China, MB0122-2) intraperitoneal injection (diluted in 0.5 mL saline, servicebio, Wuhan, China, G4702-500ML). Rats were then housed individually and allowed unrestricted access to food and water during recovery.

#### 2.2.4 Benchmark comparison under standardised resistive load

To benchmark device performance under controlled non-biological conditions, the PD-stim system was evaluated using a 100 kΩ precision resistive load and directly compared with a clinically approved tACS device (VTS-801B, Wogao Medical, Nanjing, China). Each device was independently connected to the resistive load, and output waveforms were recorded using a dual-channel oscilloscope under identical stimulation parameters.

Quantitative analysis focused on: (1) waveform fidelity, assessed by comparing recorded waveforms to the ideal sinusoidal reference, and (2) amplitude stability, determined by analysing peak-to-peak voltage variability over time.

#### 2.2.5 *In vivo* waveform recording and oscilloscope monitoring

To verify device precision under biological conditions, the implanted multi-electrode arrays were connected simultaneously to the PD-stim device for dual-site tACS delivery and to an external dual-channel oscilloscope for real-time monitoring of stimulation output. This configuration enabled direct acquisition of *in vivo* stimulation waveforms from both the medial prefrontal cortex (mPFC) and hippocampus (HPC), allowing synchronous assessment of waveform fidelity, amplitude stability, and inter-channel phase relationships.

Rats were anesthetised with 1–3% isoflurane throughout waveform recording to minimise movement artefacts and ensure stable neural impedance conditions. The two stimulation channels of the PD-stim device were connected in parallel to the implanted electrode leads and to the corresponding oscilloscope inputs using shielded connectors (wrapped with aluminium foil and connected to the ground).

To prevent external electrical interference, both the stimulation system and oscilloscope were powered by independent rechargeable lithium batteries, ensuring electrically isolated operation.

#### 2.2.6 Quantification of system fidelity

Amplitude stability was quantified by analysing the temporal variability of the peak-to-peak voltage (V_pp_) of stimulation waveforms recorded over time. For each stimulation condition, continuous voltage traces were acquired using an oscilloscope. Signals were segmented into consecutive oscillation cycles. For each cycle *i*, the peak-to-peak voltage was calculated as:

$$V_{\mathrm{pp},i}=V_{\max,i}-V_{\min,i}$$

where $V_{\max,i}$ and $V_{\min,i}$ represent the maximum and minimum voltages within a single oscillation cycle, respectively.

Amplitude stability was then assessed by computing the variability of Vpp across time:

$$\text{Amplitude Stability}=\frac{\sigma (V_{\mathrm{pp}})}{\mu (V_{\mathrm{pp}})}$$

where $\mu (V_{\mathrm{pp}})$ is the mean peak-to peak voltage across all analysed cycles (i > 5) and $\sigma (V_{\mathrm{pp}})$ is the corresponding standard deviation. Lower amplitude stability values indicate greater amplitude stability.

Signal fidelity was quantified by comparing recorded stimulation waveforms to an ideal sinusoidal reference at the commanded stimulation frequency. Voltage traces were detrended and segmented into oscillatory cycles. For each cycle, an ideal sinusoid $s(t)=A\cdot \sin(2\pi f_{0}t+\emptyset )$ was fit to the recorded signal by least squares using sine–cosine basis functions. The residual waveform r(t)=x(t)−s(t) was computed, and waveform fidelity was summarised as the cycle-integrated residual (mean absolute residual per cycle), normalised by fitted amplitude and averaged across cycles. Lower residual values indicate higher waveform fidelity.

Phase-delivery accuracy was quantified by measuring the temporal offset between dual-channel stimulation waveforms and converting this offset to a phase angle relative to the stimulation period. Dual-channel signals were detrended, band-limited around the commanded frequency (theta: 8 Hz, beta: 20 Hz, gamma: 40 Hz), and segmented into oscillatory cycles. The temporal lag between channels was estimated using peak timing and converted to phase difference ( $\Delta \emptyset =360^{{}^{\circ}}\cdot \frac{\mathrm{\Delta t}}{T}$ ). Phase-delivery accuracy was defined as the absolute deviation between measured and target phase differences and averaged across cycles:

$$\text{phase}-\text{delivery accuracy}\;(\%)=(1-\frac{|\begin{array}{l} \Delta \emptyset_{\text{measured}}-\\ \Delta \emptyset_{\text{target}} \end{array}|}{\Delta \emptyset_{\text{target}}})\times 100\%$$

#### 2.2.7 Immunohistochemistry

At the completion of experiments, rats implanted with stimulation and monitoring electrodes were euthanised with a lethal dose of isoflurane. To confirm the precise locations of implanted electrode tips, electrolytic lesions were generated by passing a 70 μA direct current for 5 s through the eletrode tips.

Animals then underwent transcardial perfusion through the left ventricle with 1 × PBS (Beyotime, Shanghai, China, CAT#: C0221A) followed by 4% paraformaldehyde (PFA, Sangon Biotech, Shanghai, CAT#: A500684). Brains were carefully removed from the skull, post-fixed in 4% PFA at 4 °C for 24 h, and subsequently transferred to 30% sucrose solution (Sinopharm, Shanghai, China, CAT#: 10021418) for cryoprotection for 48 h. Once the brains had fully sunk, coronal brain sections were cut at 100 μm thickness using a cryostat (Leica Microsystems, Wetzlar, Germany).

In addition, DiI fluorescence labeling applied to the electrode wires prior to implantation was used to further visualise electrode trajectories and final recording/stimulation sites. Brain slices were imaged using an upright fluorescence microscope (4 × objective; CX33, Olympus Corporation, Tokyo, Japan), and electrode tip positions were verified relative to standard anatomical landmarks.

#### 2.2.8 Statistical analysis

All data are presented as mean ± standard error of the mean (SEM). Statistical analyses were performed using GraphPad Prism (version 10.0) and MATLAB (2025). Normality of data distribution was assessed using the Kolmogorov–Smirnov test. If datasets met normality criteria, parametric tests were applied; otherwise, non-parametric tests were used. Statistical significance was defined as *p* < 0.05.

For between-group comparisons involving two independent groups, unpaired two-tailed Student’s t-tests were used when data were normally distributed; otherwise, Mann–Whitney U tests were applied.

## 3 Results

### 3.1 Biological impedance stability during phase-difference tACS

**Figure 1:**
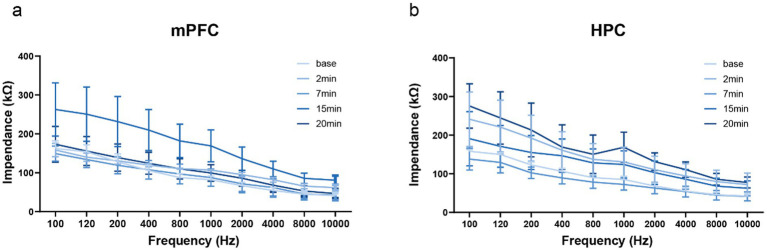
Biological impedance stability after phase-difference tACS. **(a)** Frequency-dependent impedance profiles recorded from the medial prefrontal cortex (mPFC, *n* = 3) and **(b)** the hippocampus (HPC, *n* = 3) in rats at baseline and at 2, 7, 15, and 20 min after stimulation onset. Data are presented as mean ± SEM.

During phase-difference transcranial alternating current stimulation (PD-stim), *in vivo* impedance stability was first assessed to verify the reliability of electrical coupling between the electrode and neural tissue, which provides a fundamental basis for the validity of subsequent stimulation and recording outcomes. In accordance with the stimulation and recording protocols described in the Methods section, frequency-dependent impedance was measured in the rat medial prefrontal cortex (mPFC, Figure 1a) and hippocampus (HPC, Figure 1b) throughout the stimulation period. As illustrated in Figure 1, impedance values in both brain regions decreased systematically with increasing frequency and exhibited highly consistent trajectories across multiple time points, including baseline and 2, 7, 15, and 20 min after stimulation onset. No abrupt fluctuations or time-dependent drifts were observed in either the mPFC (*n* = 3) or HPC (n = 3). These observations are consistent with the stable electrode–tissue interface and constant stimulation parameters established in the experimental design, confirming reliable electrode contact, consistent current delivery, and stable system performance throughout the entire stimulation session. Together, these findings demonstrate that the PD-stim system operates robustly under biologically relevant impedance conditions and provides a stable foundation for subsequent evaluations of waveform fidelity and phase-delivery accuracy.

### 3.2 Equivalent signal fidelity and amplitude stability between PD-stim and clinical tACS under resistive load

**Figure 2:**
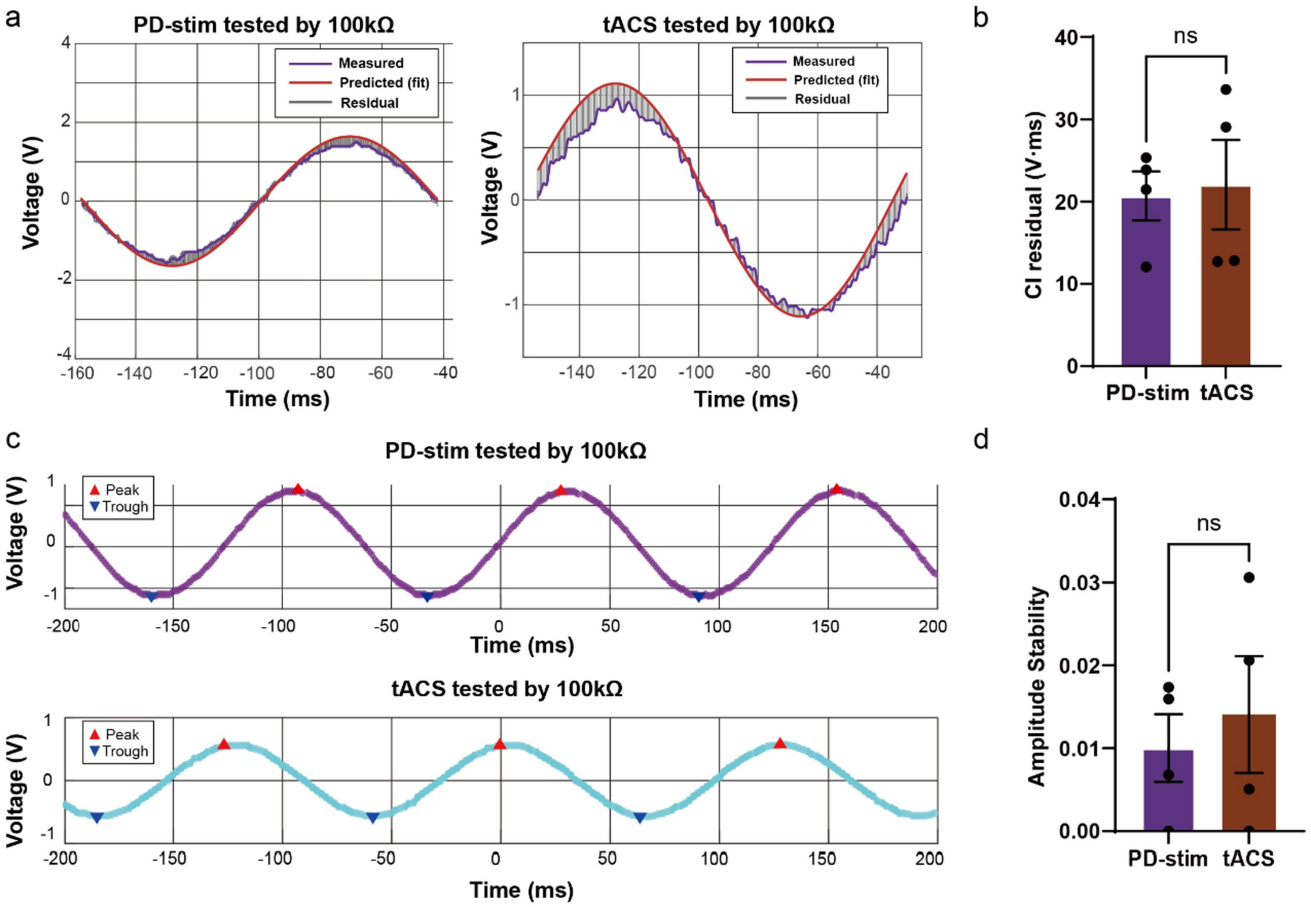
No significant difference of the signal fidelity and amplitude stability on resistance between the PD-stim device and a clinical tACS. **(a)** Representative stimulation waveforms recorded under a 100 kΩ resistive load for PD-stim (left) and a clinically approved tACS device (right). For each device, the measured waveform (blue) is shown together with the fitted ideal sinusoidal reference (red) and the residual signal (grey), illustrating waveform fidelity. **(b)** Quantitative comparison of signal fidelity between PD-stim and clinical tACS, expressed as the cycle-integrated (CI) residual. No significant difference was observed between devices (unpaired two-sided *t*-tes*t*, *t* = 0.2213, *p* = 0.8322; mean ± SEM: 1.372 ± 6.202, PD-stim: *n* = 4, tACS: *n* = 4). **(c)** Representative traces illustrating peak and trough detection used for peak-to-peak voltage (Vpp) calculation in PD-stim (top) and clinical tACS (bottom) recordings under resistive load conditions. **(d)** Comparison of waveform amplitude stability between PD-stim and clinical tACS, quantified as the coefficient of variation of Vpp across cycles (mean ± SEM). No significant difference was detected between devices (unpaired two-sided *t*-test, *t* = 0.4982, *p* = 0.6361; mean ± SEM: 0.004052 ± 0.008133, PD-stim: *n* = 4, tACS: *n* = 4). Each dot represents an individual recording. ns, Not significant.

To benchmark device performance under controlled non-biological conditions, PD-stim was directly compared with a clinically approved tACS device (VTS-801B, Wogao Medical, Nanjing, China) using a 100 kΩ resistive load. Representative waveforms from both devices closely matched the fitted ideal sinusoidal reference, with minimal residual error (Figure 2a). Quantitative analysis of signal fidelity, expressed as the cycle-integrated (CI) residual, revealed no significant difference between PD-stim and clinical tACS (unpaired two-sided *t*-test, *t* = 0.2213, *p* = 0.8322; Figure 2b).

Similarly, waveform amplitude stability, quantified as the coefficient of variation of peak-to-peak voltage (Vpp) across oscillatory cycles, did not differ significantly between devices (Figure 2d), and representative traces illustrating peak and trough detection used for Vpp calculation are shown in Figure 2c.

### 3.3 High waveform fidelity and amplitude stability *in vivo* in mPFC and HPC

**Figure 3:**
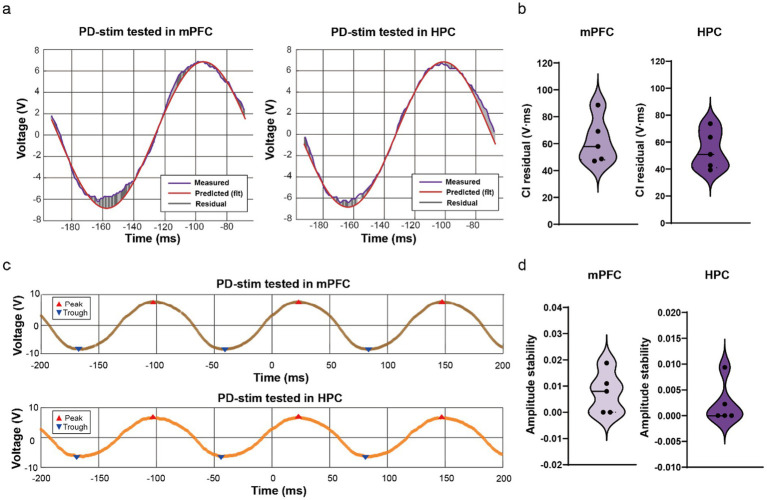
The signal fidelity and amplitude stability tested in the prefrontal and hippocampus regions of rats using the PD-stim device. **(a)** Representative stimulation waveforms recorded *in-vivo* from the mPFC (left) and HPC (right) during PD-stim delivery. For each region, the measured waveform (blue) is shown together with the fitted ideal sinusoidal reference (red) and the residual signal (grey), illustrating high waveform fidelity under biological neural load conditions. **(b)** Quantification of signal fidelity in mPFC (left, *n* = 5) and HPC (right, *n* = 5), expressed as the CI residual. Each dot represents an individual recording, and violin plots illustrate the distribution of residual values across animals. **(c)** Representative voltage traces illustrating peak and trough detection used for Vpp calculation in mPFC (top) and HPC (bottom) recordings. **(d)** Quantification of waveform amplitude stability in mPFC (left, *n* = 5) and HPC (right, *n* = 5), expressed as the coefficient of variation of Vpp across oscillatory cycles. Each dot represents an individual recording, and violin plots illustrate the distribution of amplitude stability values across animals.

To validate device performance under biological neural load, waveform fidelity and amplitude stability were further evaluated using implanted electrodes in rats. Representative in vivo recordings from both mPFC and HPC demonstrated close correspondence between measured waveforms and fitted sinusoidal references, with minimal residual deviation (Figure 3a).

Quantitative analysis confirmed high signal fidelity in both regions, as reflected by low CI-residual values in mPFC (*n* = 5) and HPC (*n* = 5), with consistent distributions across animals (Figure 3b). Likewise, amplitude stability remained high in both regions, with low coefficients of variation of Vpp across cycles and narrow distribution ranges (Figures 3c,d). Together, these results demonstrate that PD-stim maintains stable, high-quality stimulation waveforms under biologically realistic conditions in distributed brain targets.

### 3.4 Accurate and consistent phase-difference delivery across frequencies

**Figure 4:**
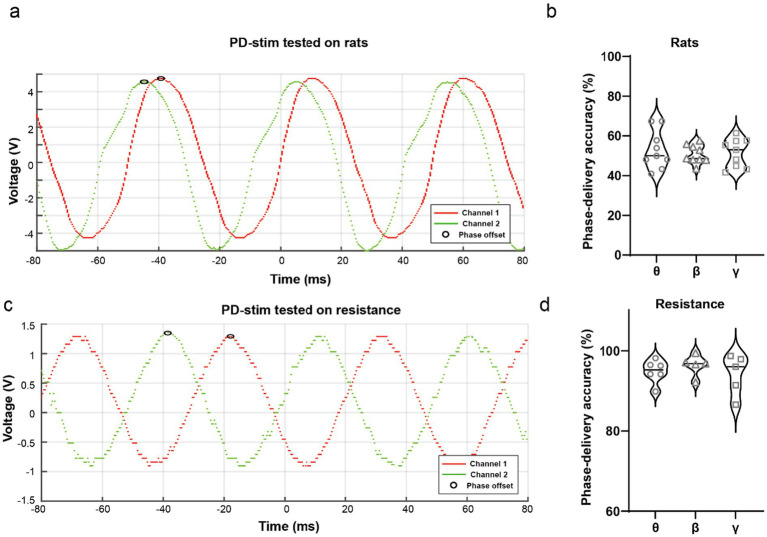
Dual-channel waveforms and phase-delivery accuracy of the PD-stim system. **(a)** Representative dual-channel stimulation waveforms recorded *in vivo* in rats, showing Channel 1 (red) and Channel 2 (green) outputs with a stable and predefined phase offset. Open circles indicate corresponding phase reference points used to calculate inter-channel temporal offsets. **(b)** Quantification of phase-delivery accuracy in rats across theta (*θ*, 8 Hz, *n* = 9), beta (*β*, 20 Hz, *n* = 9), and gamma (*γ*, 40 Hz, *n* = 9) stimulation conditions. Violin plots illustrate the distribution of phase-delivery accuracy values, with individual data points representing separate recordings. **(c)** Representative dual-channel stimulation waveforms recorded under a 100 kΩ resistive load, demonstrating stable sinusoidal outputs and preserved phase offsets between channels in a controlled non-biological environment. **(d)** Quantification of phase-delivery accuracy under resistive load conditions across theta (θ, 8 Hz, *n* = 5), beta (β, 20 Hz, *n* = 5), and gamma (γ, 40 Hz, *n* = 5) stimulation conditions. Violin plots display the distribution of accuracy values, confirming consistent phase-difference delivery across frequencies and testing environments.

A defining feature of PD-stim is its ability to deliver precise and programmable phase differences between stimulation channels. Dual-channel recordings obtained *in vivo* demonstrated stable sinusoidal waveforms with clearly defined inter-channel phase offsets (Figure 4a). Quantification of phase-delivery accuracy in rats revealed consistent performance across theta (8 Hz, *n* = 9), beta (20 Hz, *n* = 9), and gamma (40 Hz, *n* = 9) frequency conditions (Figure 4b).

To further assess phase accuracy under controlled conditions, the same analyses were performed using a 100 kΩ resistive load. Representative waveforms confirmed preserved phase offsets between channels (Figure 4c), and quantitative results demonstrated high phase-delivery accuracy across theta (*n* = 5), beta (*n* = 5), and gamma (*n* = 5) stimulation frequencies (Figure 4d). Importantly, phase accuracy was consistent across biological and non-biological testing environments, indicating robust phase control independent of load conditions.

## 4 Discussion

In this study, we engineered and systematically validated a novel phase-difference transcranial alternating current stimulation (PD-stim) platform, purpose-built to execute highly precise, stable, and programmable dual-site neuromodulation. Through a rigorous hierarchy of bench-top and *in vivo* testing, we demonstrated that the PD-stim architecture achieves (i) robust impedance stability within living neural tissue, (ii) waveform fidelity and amplitude constancy that parallel clinical-grade tACS standards under both standardized resistive and complex biological loads, and (iii) unprecedented phase-delivery accuracy across multiple frequency bands. Collectively, these results validate PD-stim as a highly reliable, phase-specific tool optimized for network-targeted neuromodulation.

### 4.1 Mechanistic context: overcoming impedance-driven instability

A major challenge for non-invasive and invasive neuromodulation systems is maintaining stable current delivery in the presence of dynamic biological impedance (Satzer et al., 2020). Brain tissue impedance varies across regions, frequencies, and time, and instability can compromise waveform integrity and stimulation safety (Cui et al., 2024). Our *in vivo* measurements in both the medial prefrontal cortex and hippocampus demonstrate stable, time-dependent impedance profiles during stimulation, indicating reliable electrode–tissue coupling and effective current control. This validates the efficacy of our high-compliance, constant-current regulation in preserving reliable electrode–tissue coupling. Such biological robustness is an absolute prerequisite for dual-site neuromodulation, where the precise spatiotemporal preservation of both relative phase timing and amplitude consistency across distributed targets is non-negotiable.

### 4.2 Field context: advancing phase-specific neuromodulation

To establish translational relevance, we benchmarked the PD-stim system against a clinically approved tACS device. Under a standardized 100 kΩ resistive load—a widely accepted proxy for biological impedance—PD-stim exhibited equivalent waveform fidelity and amplitude stability. Crucially, this high-fidelity performance seamlessly translated to *in vivo* conditions, as verified by direct intracranial recordings. These findings underscore that the PD-stim architecture meets stringent clinical-grade standards for fundamental waveform quality, even while executing the computationally demanding task of high-resolution phase-difference control. Achieving this dual capability without penalizing output stability represents a critical engineering leap.

The defining innovation of the PD-stim platform is its capacity to dynamically program and maintain highly accurate inter-channel phase differences—a capability fundamental to the exogenous modulation of communication across distributed neural networks (Fries, 2015). Through simultaneous dual-channel recordings, we verified that PD-stim consistently sustains pure sinusoidal waveforms with rigidly locked phase offsets, regardless of whether it operates against a resistive load or within living tissue. Quantitative analysis further confirmed exceptional phase-delivery accuracy across the theta, beta, and gamma frequency bands. This degree of temporal precision far surpasses the validation thresholds typical of conventional tACS systems, directly resolving a major technological bottleneck in contemporary neuromodulation.

Physiological neural communication is mechanistically predicated on the precise relative timing of oscillatory activity between interacting brain regions (Samantzis et al., 2025). Correspondingly, the pathological breakdown of these inter-regional phase relationships is a primary signature of age-related neurological disorders, including stroke and progressive neurodegenerative diseases (Al-Ezzi et al., 2024; Rogala et al., 2024; Tscherpel et al., 2025). In this context, the present study is intentionally positioned as a foundational engineering validation, focusing on device performance rather than functional or therapeutic outcomes. The demonstrated ability of PD-stim to reliably control inter-regional phase relationships provides a robust technical basis for future mechanistic investigations of network-level dynamics. By enabling controlled, phase-specific stimulation paradigms, PD-stim offers a versatile experimental tool to probe the causal roles of oscillatory synchrony in brain function, with potential relevance for future translational research.

### 4.3 Limitations and future directions

Several limitations outline the trajectory for future development. First, direct benchmarking of phase-delivery accuracy against existing clinical tACS devices under resistive loads was unfeasible, primarily because conventional systems intrinsically lack independent, high-resolution phase-control modules. Second, while our invasive intracranial recordings provide the ultimate ground-truth validation for waveform and phase precision, subsequent studies must translate these assessments into non-invasive human paradigms, which will necessitate accounting for the complex signal attenuation caused by the skull and scalp. Finally, although this study firmly establishes the system’s engineering accuracy and electrophysiological stability, it does not evaluate functional neuromodulation effects, including behavioral, cognitive, or long-term neuroplastic outcomes. As such, no conclusions can be drawn regarding efficacy at the systems or behavioural level. In addition, the *in vivo* validation was conducted in an anaesthetised rodent model, which does not fully capture the complexity of neural dynamics in awake or human conditions. Furthermore, the relatively modest sample sizes used in the present study warrant cautious interpretation and should be expanded in future work. Addressing these limitations will require dedicated, model-guided experimental designs and subsequent validation in larger cohorts and translational settings.

## 5 Conclusion

In summary, we present a rigorously validated phase-difference tACS system capable of delivering stable, high-fidelity, and phase-accurate dual-channel stimulation under both biological and controlled testing conditions. PD-stim overcomes key technical limitations of existing neuromodulation devices and provides a robust engineering platform for future studies investigating phase-specific modulation of distributed brain networks. While the present work does not address functional or therapeutic outcomes, it establishes a technical foundation with potential relevance for subsequent mechanistic and translational research.

## Data Availability

The original contributions presented in the study are included in the article/supplementary material, further inquiries can be directed to the corresponding authors.
